# Retinal Ganglion Cell Survival and Axon Regeneration after Optic Nerve Injury: Role of Inflammation and Other Factors

**DOI:** 10.3390/ijms231710179

**Published:** 2022-09-05

**Authors:** Kimberly A. Wong, Larry I. Benowitz

**Affiliations:** 1 Department of Neurosurgery, Boston Children’s Hospital and Harvard Medical School, Boston, MA 02115, USA; 2 F.M. Kirby Neurobiology Center, Boston Children’s Hospital, Boston, MA 02115, USA; 3 Department of Ophthalmology, Harvard Medical School, Boston, MA 02115, USA

**Keywords:** retina, inflammation, transcription, CNS repair, optic nerve, oncomodulin, myeloid cells, regeneration

## Abstract

The optic nerve, like most pathways in the mature central nervous system, cannot regenerate if injured, and within days, retinal ganglion cells (RGCs), the neurons that extend axons through the optic nerve, begin to die. Thus, there are few clinical options to improve vision after traumatic or ischemic optic nerve injury or in neurodegenerative diseases such as glaucoma, dominant optic neuropathy, or optic pathway gliomas. Research over the past two decades has identified several strategies to enable RGCs to regenerate axons the entire length of the optic nerve, in some cases leading to modest reinnervation of di- and mesencephalic visual relay centers. This review primarily focuses on the role of the innate immune system in improving RGC survival and axon regeneration, and its synergy with manipulations of signal transduction pathways, transcription factors, and cell-extrinsic suppressors of axon growth. Research in this field provides hope that clinically effective strategies to improve vision in patients with currently untreatable losses could become a reality in 5–10 years.

## 1. Introduction

Restoring vision after optic nerve injury requires maintaining retinal ganglion cell (RGC) survival while enabling these cells to re-extend axons and re-establish connections in appropriate target areas of the brain. Achieving these goals will entail suppressing pathological processes that lead to RGC death, restoring an active growth state to these neurons, and ensuring that growing axons navigate back to visual relay nuclei of the di- and mesencephalon. One area we emphasize here is the surprising role of the innate immune system in modulating RGC survival, axon regrowth, and myelination. Understanding the mechanisms that underlie these phenomena may eventually enable us to regulate the inflammatory response that inevitably accompanies CNS damage to our advantage and/or isolate beneficial immune-derived factors to improve functional recovery after optic neuropathy. We will also briefly review other strategies to promote RGC survival and regeneration that, in many cases, act synergistically with manipulations of the innate immune system. We are not covering important recent developments in replacing lost RGCs with embryonic stem cells or pluripotent stem cells, though these approaches will require the new neurons to extend axons to appropriate brain areas and therefore involve some of the same issues as axon regeneration from injured RGCs. 

## 2. History

Unlike neurons in the peripheral nervous system (PNS), those of the central nervous system (CNS) cannot spontaneously regenerate damaged axons, and, consequently, CNS injuries or various neurodegenerative diseases often result in severe and irreparable functional losses. The seminal studies of Tello and his mentor, Santiago Ramon y Cajal, were the first to show that, under certain experimental conditions, CNS neurons can be induced to regenerate damaged axons. These early 20th century studies showed that, following optic nerve transection, RGCs could regrow axons through a peripheral nerve graft implanted at the site of axotomy [1], implying that, although these neurons remain regeneration-competent, conditions that enable regeneration to occur are not normally present in the CNS environment after injury. Beginning several decades later, the scientific community undertook hundreds of studies to understand the factors that prevent or enable neurons to regenerate injured axons. Here, we first summarize the results of our group and others to understand how inflammation can be harnessed to promote RGC neuroprotection and enable these neurons to regenerate axons, then proceed to describe complementary lines of investigation. 

## 3. Intraocular Inflammation Promotes RGC Survival and Axon Regeneration

**Figure 1:**
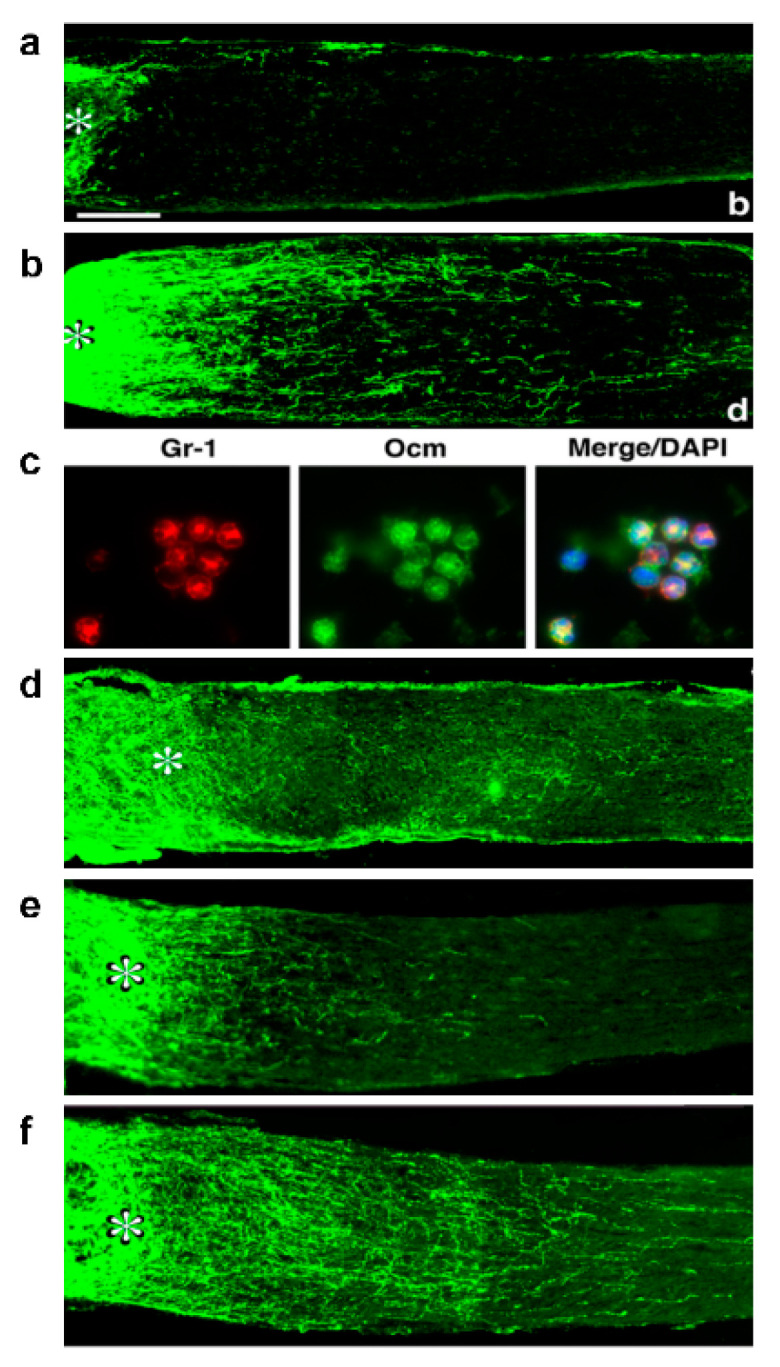
Inflammation-induced optic nerve regeneration and the role of Oncomodulin (Ocm). (**a**) Negative control: Absence of axon regeneration following optic nerve injury (asterisk: site of optic nerve crush injury). (**b**) Intraocular inflammation induced by injury to the lens (shown here) or by other means cited in the text enables retinal ganglion cells (RGCs) to regenerate axons past the injury site. (**c**) Intraocular inflammation is associated with a rapid infiltration of Gr-1-positive neutrophils (red) that express high levels of Ocm (green). (**d**) P1, a peptide antagonist of Ocm based on the N-terminus of the protein, suppresses Zymosan-induced regeneration. (**e**) Slow-release polymer beads containing the cAMP analog CPT-cAMP induce minimal regeneration after optic nerve injury. (**f**) Extensive regeneration with slow-release beads containing Ocm + CPT-cAMP [5,6,13].

Although the retina, like other parts of the CNS, was long thought to be “immune privileged”, i.e., resistant to inflammation, many studies now show that neuroinflammation plays a critical role in the pathology of chronic ocular diseases. In glaucoma and ocular auto-immune diseases, chronic activation of tissue-resident cells (microglia, Muller glia, and astrocytes) can augment neurodegeneration by stimulating the adaptive immune system to target ocular tissues [2,3,4]. Paradoxically, however, inducing sterile inflammation in the eye in animal models augments RGC survival and axon regeneration. Our lab inadvertently discovered that injury to the lens enhances the survival of RGCs after optic nerve injury (ONI) and enables these cells to begin regenerating axons well beyond the injury site. Lens injury (LI) leads to the infiltration of neutrophils and macrophages that express factors that increase RGC survival and cause RGCs to up-regulate genes linked to axon growth and regeneration (Figure 1a,b [5]). These effects can be mimicked by intravitreal injection of Zymosan, a yeast cell wall preparation [6,7] that activates Toll-like receptor 2 (TLR2) and the pattern recognition receptor dectin-1. Accordingly, the effects of LI or Zymosan are suppressed by blocking receptors for the pro-inflammatory chemokines CCL2 [8] or CXCL5 [9], mimicked by the TLR2 agonist Pam3Cys [10] or the Dectin-1 agonist b-glucan [11], and exceeded by injecting a newly defined set of immature neutrophils [12]. These findings raise the possibility that defined signals associated with sterile inflammation might serve as safe biologics to promote neuroprotection and axon regeneration without the deleterious effects of intraocular inflammation. 

## 4. Oncomodulin: A Key Mediator of Inflammation-Induced Regeneration

The first myeloid cell-derived protein found to play a key role in inflammation-induced regeneration is the 11 kDa Ca^2+^-binding protein Oncomodulin (Ocm) [13]. Stimulating ocular inflammation via LI or intravitreal Zymosan injection elicits a massive influx of Ocm-expressing neutrophils into the vitreous (Figure 1c) and elevation of Ocm in the inner retina [5,13,14,15]. Ocm binds with high affinity to a cell-surface receptor on RGCs in a cAMP-dependent manner (Kd ~30 nM) [16] and, in the presence of a cAMP analog (or forskolin) plus D-mannose, enhances neurite outgrowth in cultured RGCs well beyond levels stimulated by well-established growth factors such as brain-derived neurotrophic factor (BDNF), fibroblast growth factor-2 (FGF2), ciliary neurotrophic factor (CNTF), or glial cell-derived neurotrophic factor (GDNF) [13]. In loss-of-function studies, a function-blocking anti-Ocm antibody or peptide antagonist of Ocm nearly eliminates inflammation-induced regeneration (Figure 1d) [5,13,14], while conversely, slow release of Ocm and a cAMP analog from polymer beads stimulates strong regeneration (Figure 1e,f) [15]. However, later studies showed that the beads alone induce some inflammation that contributes to these effects, implying that additional inflammation-associated factors complement the effects of Ocm in vivo. 

## 5. Macrophage-Derived SDF1 Complements the Effects of Ocm

**Figure 2:**
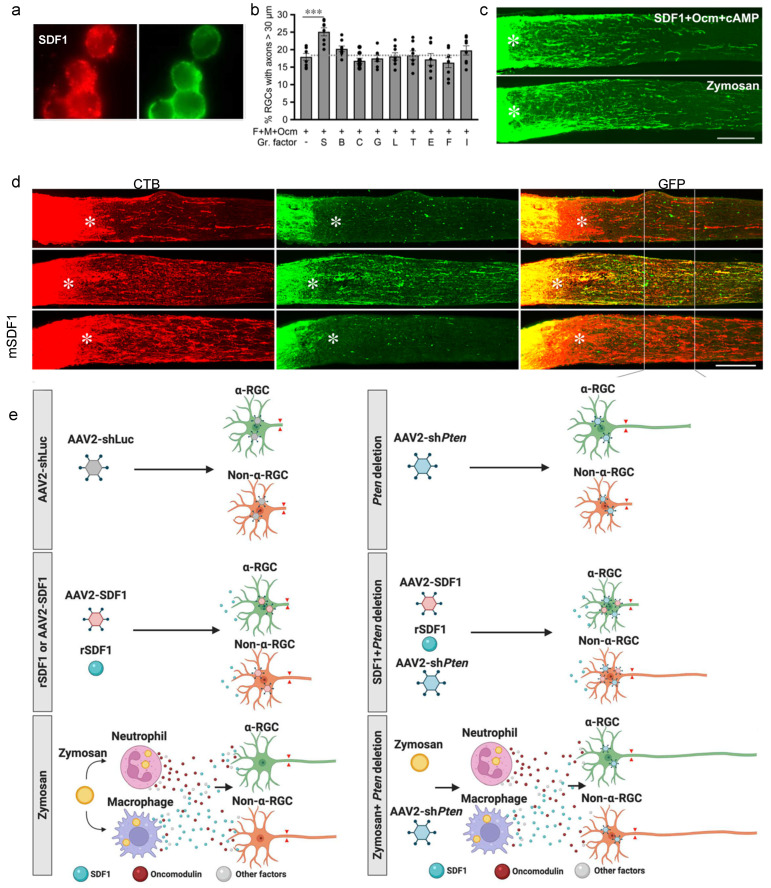
Macrophage-derived SDF1 complements the effects of Ocm (previous page). (**a**) SDF1 (red) is highly expressed in F4/80 macrophages that infiltrate the vitreous by 24 h after intraocular injection of Zymosan. (**b**) Among multiple trophic factors tested in dissociated adult retinal cell cultures, SDF1 is the only factor that enhances the effects of Ocm (combined with co-factors mannose and CPT-cAMP). B: Brain-derived neurotrophic factor (BDNF); C: ciliary neurotrophic factor (CNTF); G: glial cell-derived trophic factor (GDNF); L: leukemia inhibitory factor (LIF); T: tumor-necrosis factor (TNF); F: fibroblast growth factor-2; I: insulin-like growth factor 2 (IGF2). *** *p* < 0.001. (**c**) SDF1 combined with Ocm and CPT-cAMP induces similar levels of regeneration as Zymosan. (**d**) SDF1 alters the response of different RGC populations to PTEN deletion. aRGCs, the population that extends axons in response to Pten deletion, are identified by virtue of expressing GFP from the Kcng4 promoter. Axons arising from all RGCs, whether aRGCs or non-aRGCs, are labeled with CTB. Top row: In response to SDF1 alone, regenerating GFP-negative axons all arise from non-aRGCs. Middle row: Pten deletion alone induces regeneration primarily from aRGCs (note extensive overlap of GFP and CTB in last panel). Bottom row: Combining SDF1 and Pten deletion suppresses regeneration from aRGCs while strongly increasing overall levels of regeneration from non-αRGCs. (**e**) Schematic illustration showing the response of different RGC populations to various treatments. Top row: AAV2 expressing anti-Pten shRNA (AAV2-shPten) induces axon growth primarily from aRGCs (green cells). AAV2-shLuciferase virus (AAV2-shLuc) has no effects. Middle row: Either recombinant SDF1 (rSDF1) or AAV2 expressing SDF1 (AAV2-SDF1) induces moderate axon growth primarily from non-aRGCs (middle left: orange cells). When combined with Pten deletion, SDF1 induces non-aRGCs to regenerate lengthy axons but prevents aRGCs from responding to Pten deletion (middle right). Bottom row: Zymosan elevates levels of neutrophil-derived Ocm, macrophage-derived SDF1, and other factors, stimulating regeneration from both α- and non-αRGCs (bottom left); Pten deletion combined with Zymosan strongly augments outgrowth from both subtypes (bottom right) [8]. Asterisks show lesion site.

Subsequent studies found that stromal cell-derived factor 1 (SDF1, CXCL12) is highly expressed by infiltrative macrophages following intraocular inflammation (Figure 2a) and complements the effects of Ocm [8]. In cell culture, stimulation with SDF1 induces moderate axon outgrowth from RGCs, and, importantly, is the only factor among many tested (e.g., CNTF, BDNF, FGF2, GDNF, others) that enhances the effects of Ocm (Figure 2b) [8]. In vivo, Zymosan-induced axon regeneration and RGC survival are suppressed by either deleting SDF1 in myeloid cells (LysM-Cre: CXCL12^f/f^ mice), deleting the primary SDF1 receptor, CXCR4, in RGCs (intraocular injection of adeno-associated virus seroform 2 expressing Cre recombinase in CXCR4^f/f^ mice), or inhibiting this pathway with the CXCR4 antagonist, AMD3100 [8]. Conversely, SDF1 combined with Ocm and a cAMP analog induces as much regeneration and neuroprotection as Zymosan (Figure 2c), thus demonstrating the sufficiency of defined molecules in promoting regeneration. 

Prior work showed that deleting the tumor-suppressor gene Pten in RGCs induces considerable axon regeneration that primarily arises from aRGC [17]. In contrast, SDF1 stimulates outgrowth in non-aRGCs and enables them to respond strongly to Pten deletion but paradoxically suppresses aRGCs’ response to Pten deletion [8]. Zymosan, which elevates levels of both SDF1 and Ocm, stimulates outgrowth from aRGCs and non-aRGCs and amplifies the effects of Pten deletion for multiple RGC classes (Figure 2e). 

## 6. CNTF Gene Therapy, like Zymosan and LI, Promotes Regeneration via Neuroinflammation

**Figure 3:**
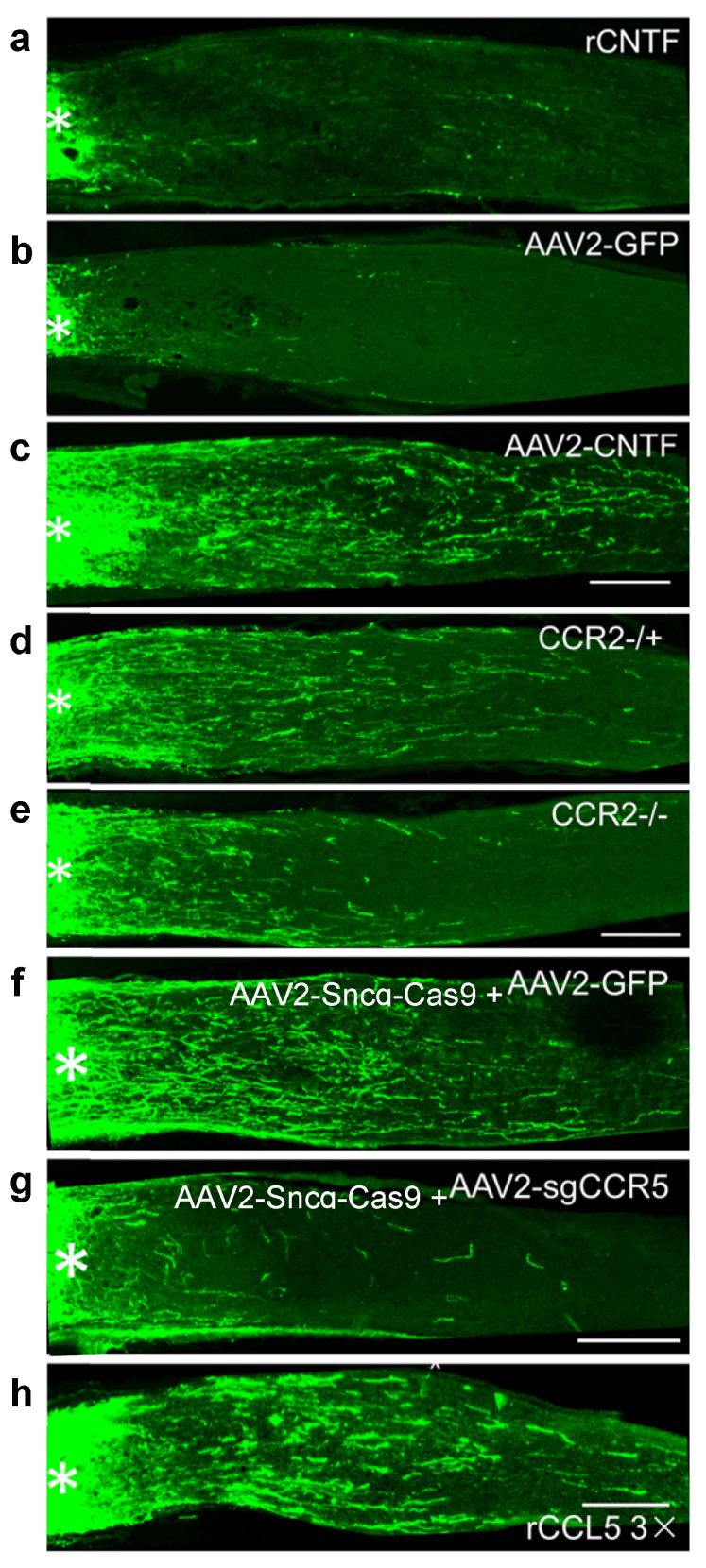
CNTF gene therapy induces optic nerve regeneration by augmenting inflammation and chemokine CCL5. (**a**) Recombinant CNTF protein (1 µg) does not induce appreciable regeneration. (**b**,**c**) Unlike a control adeno-associated protein expressing green fluorescent protein (AAV2-GFP), AAV2 expressing CNTF induces appreciable levels. (**d**,**e**) Whereas mice heterozygous for the chemokine 2 receptor (CCR2^+/−^) regenerate axons in response to CNTF gene therapy (**d**), homozygous null mice show a strongly diminished response (**e**). (**f**,**g**) CRISPR-Cas9 mediated deletion of the chemokine 5 receptor CCR5. Mice received intraocular injection of an adeno-associated virus expressing Cas9 driven by the synuclein-g promoter plus a second virus expressing either GFP ((**f**), control) or a small guide RNA directed to CCR5 (**g**), which nearly eliminated regeneration induced by CNTF gene therapy. (**h**) Recombinant CCL5 induces nearly as much regeneration as CNTF gene therapy. Asterisks indicate lesion site [22].

CNTF is a leading candidate for neuroprotection in several ocular diseases and has also been of considerable interest for optic nerve regeneration. Although one group has maintained that CNTF (and/or LIF) is the major mediator of inflammation-induced regeneration [18,19], our lab and others found that recombinant CNTF (rCNTF) has little or no effect on optic nerve regeneration (Figure 3a) unless SOCS3, a suppressor of the Jak-STAT signaling pathway, is deleted from RGCs [7,20,21,22,23]. On the other hand, many studies have found that CNTF gene therapy (i.e., adeno-associated virus AAV2 expressing CNTF) induces robust regeneration (Figure 3c) [22,24,25,26,27,28,29,30,31]. We discovered that CNTF gene therapy induces far more intraocular inflammation than rCNTF due to baseline inflammation associated with intraocular viral vectors [32] combined with the chemotactic effect of CNTF in amplifying this inflammation [33,34]. The effects of CNTF gene therapy are almost completely lost in mice lacking CCR2, a receptor involved in macrophage recruitment and polarization (Figure 3d,e), and in mice lacking neutrophils [22]. Further work identified the chemokine CCL5 acting on its cognate receptor, CCR5, as the primary mediator of the neuroprotective and pro-regenerative effects of CNTF gene therapy, with Ocm and SDF1 playing lesser roles. Deletion of CCR5 in RGCs or the CCR5 antagonist DAPTA strongly suppress the effects of CNTF gene therapy (Figure 3f,g), whereas recombinant CCL5 mimics these effects (Figure 3h). Thus, these results position CCL5 as another candidate therapy for optic nerve repair and perhaps other optic neuropathies [22]. 

## 7. Inflammatory Pre-Conditioning Enables Robust Axon Regeneration

Inducing LI 2 weeks prior to optic nerve injury increases regeneration to a far greater extent than LI or Zymosan applied at the time of nerve injury or by Zymosan preconditioning [35]. Repeated episodes of LI prior to and again after nerve injury transforms neurons into a strong growth state, enabling many RGCs to extend axons the full length of the optic nerve within a few weeks. This effect requires monocyte infiltration into the eye, whereas microglia, neutrophils, and T-cells are not required, nor does it depend upon any of the inflammation-induced growth factors that we have identified to date (Ocm, SDF1, CCL5: Feng et al., in preparation).

## 8. Synergy between Intraocular Inflammation and Counteracting Cell-Extrinsic Suppressors of Axon Growth

**Figure 4:**
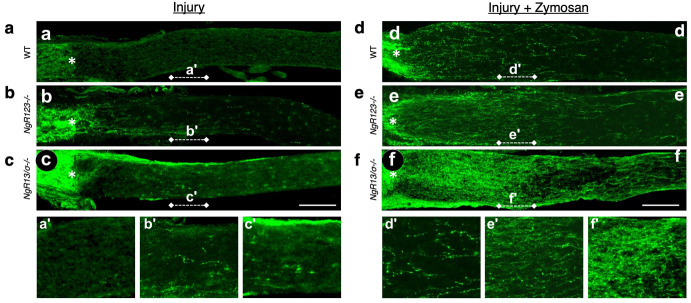
Synergy between intraocular inflammation and deleting receptors for cell-extrinsic suppressors of axon growth. (**a**–**f**) Longitudinal sections through the optic nerves of wild-type mice (WT) or mice lacking all 3 isoforms of the Nogo receptor (NgR123^−/−^) or of NgR1 and 3 plus PTPs, a receptor that mediates inhibitory effects of chondroitin sulfate proteoglycans (CSPGs). As indicated, mice either underwent optic nerve injury alone or with intraocular inflammation following intraocular injection of zymosan. (**a’**–**f’**) Regions of optic nerves shown at greater magnification in the corresponding panels below. Note the dramatic increase in regeneration when combining intraocular inflammation with deletion of receptors for the inhibitory molecules associated with myelin and CSPGs. Asterisks show lesion site [40].

Following nerve injury, myelin debris, the scar that forms at the injury site, and axon-repellant guidance cues all suppress axon regeneration in the optic nerve and elsewhere in the CNS [36,37,38]. Axon regeneration induced by intraocular inflammation is augmented several-fold by deleting all isoforms of NgR, the receptor expressed on axons and nerve terminals that mediates growth-suppressive effects of the myelin-associated proteins oligodendrocyte-myelin glycoprotein (OMgp), myelin-associated glycoprotein (MAG), and, most potently, isoforms of the protein Nogo (Figure 4). Similarly synergistic effects are seen by combining intraocular inflammation with suppression of signaling through the small GTPase RhoA, which is intracellularly activated by growth-inhibitory signals, or by deleting PTPs, a receptor that mediates growth-suppressive effects of chondroitin sulfate proteoglycans (CSPGs) [39,40,41,42], or by enzymatically blocking the effects of chondroitin sulfate proteoglycans (CSPGs) [43]. Combined deletion of two isoforms of NgR plus PTPs results in extraordinary levels of regeneration when coupled with Zymosan-induced inflammation (Figure 4f) [40]. Axon regeneration can also be augmented by increasing inflammation within the optic nerve, presumably by increasing phagocytosis of myelin debris and elements of the fibrotic scar that forms at the injury site [44].

## 9. Role of Microglia in RGC Survival and Axon Regeneration

**Figure 5:**
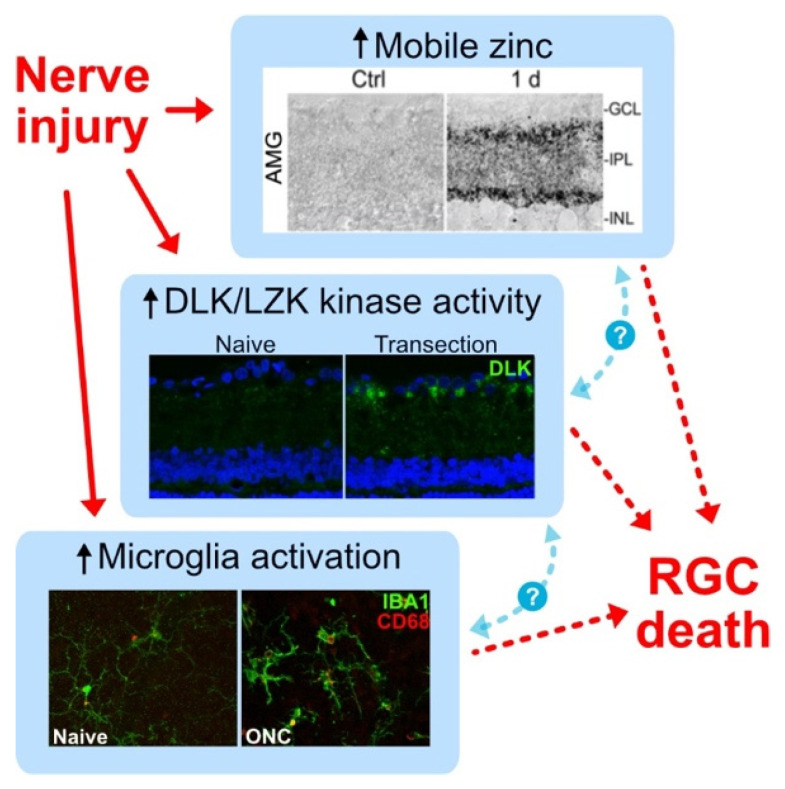
Injury-induced pathways contributing to RGC death after optic nerve injury. Encircled question marks (?) indicate as yet unknown relationships. Up arrows indicate increases.

Microglia are the resident immune cells of the nervous system, and together with Mueller glia and astrocytes, they help maintain retinal homeostasis. Microglia are concentrated in the inner and outer plexiform layers of the retina and throughout the optic nerve, with long ramified processes that interact with and surveil the neural environment and regulate retinal synapses [45,46]. Non-homeostatic (or “reactive”) microglia are characteristic of many neurodegenerative diseases, which, in the eye, include glaucoma [46,47,48,49], and photoreceptor degeneration [50,51]. It remains unclear whether a chronic microglial response is ultimately beneficial or harmful or whether the microglial response is modulated by other injury-induced signals, including the elevation of zinc or activation of the DLK and LZK kinase cascades in RGCs (Figure 5).

Elevation of mobile zinc in the inner retina, activation of the DLK and LZK kinase cascades in RGCs, and reaction of resident microglia are hallmarks of optic nerve injury and have all been shown to modulate RGC death. However, mechanistic interactions among these three pathways remain unknown.

Animal studies indicate that reducing microglial activation in glaucoma and other CNS models reduces neuronal cell death and helps resolve neuroinflammation [52,53,54,55], supporting the idea that chronic neuroinflammation is harmful in CNS disease. Following optic nerve damage and in glaucoma, one proposed mechanism for RGC death is that reactive microglia express proteins that include tumor necrosis factor-⍺ (TNF⍺), interleukin 1⍺ (IL-1⍺), and the complement protein C1q, which together polarize astrocytes to a pro-inflammatory “A1” state [56,57,58,59]. A1 astrocytes can in turn promote synapse degradation and induce neuron and oligodendrocyte cell death by secreting long-chain saturated lipids APOE and APOJ-containing lipoparticles [46,56,57,58,59,60,61,62]. Although blocking A1 polarization is reported to be strongly neuroprotective after ONI and in mouse glaucoma models [56,58], it is unlikely that microglia are the sole source of A1-inducing proteins. Retinal Tnfa, Il1a, and C1qa expression are all upregulated 3–5 days after optic nerve injury, which correlates with the response timeline of microglia [63]; yet microglial depletion (with the Colony stimulating factor 1 receptor inhibitor PLX5622) does not substantially reduce Tnfa levels. These findings suggest that other cells such as Muller glia or astrocytes may play a role [64,65,66,67]. As TNF⍺ signaling mediates RGC and oligodendrocyte death in animal models of glaucoma, likely by increasing the expression of Fas ligand [4,68,69], sustained Tnfa expression provides a plausible explanation for why microglial deletion is insufficient to provide neuroprotection [58,63,70].

One possible contributor to microglial activation in the retina is the elevation of mobile zinc (Zn^2+^). After ONI, Zn^2+^ accumulates in presynaptic boutons of amacrine cells from which it is exocytosed, contributing to RGC death and repression of regeneration after ONI [71]. In cell culture, the elevation of extracellular Zn^2+^ exacerbates microglial activation to a pro-inflammatory M1 state, resulting in increased nitric oxide (NO) production and altered cytokine expression [72,73,74,75]. In vivo, reducing extracellular Zn^2+^ via intraocular Zn^2+^ chelators or genetic knockout of the vesicular Zn^2+^ transporter ZnT3 reduces microglial activation after ONI [63,71], suggesting that Zn^2+^ elevation in the retina may be one factor contributing to the microglial response after ONI.

**Figure 6:**
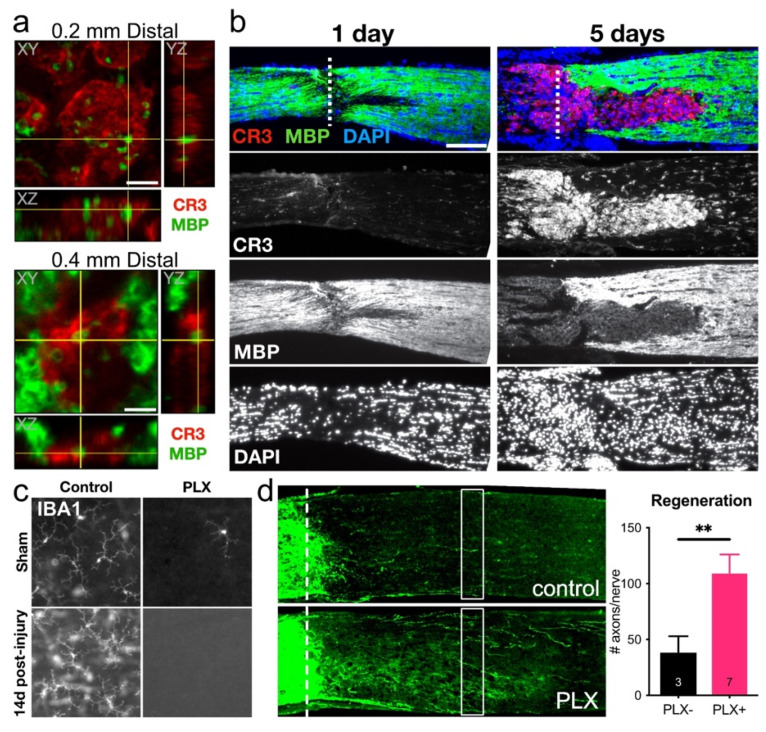
Phagocytic microglia/monocytes alter the local environment of the injury site and modulate axon regeneration. (**a**) Single confocal planes with orthogonal views of myelin basic protein (MBP) debris inside CR3+ cells. Images are from 0.2 and 0.4 mm distal to the injury site at 14 DPI [44]. (**b**) CR3+ microglia and monocytes expand within the site of injury (dotted line) from 1 to 5 days after crush, resulting in a progressive clearance of MBP from the distal optic nerve, allowing for uninhibited axon regeneration [44]. (**c**) Treatment with CSF1R inhibitor, PLX5622 (PLX) results in efficient clearance of IBA1+ microglia from the retina, which is maintained by 14 days after ONI (14d post-ONI). (**d**) Microglia deletion enhances the number of GAP43+ (green) axons regenerating past the crush site (dotted line). Right: Quantitation of regenerating axons 0.5 mm distal to the injury site (☐); mean ± s.e.m, n = optic nerves, **, *p* ≤ 0.01 by *t*-test [44].

Yet, despite the well-documented negative aspects of microglial activation, microglia can play a beneficial role after optic nerve injury. These latter effects are mediated through complement pathway-induced activation of resident microglia and/or by increasing the infiltration of CR3-expressing monocytes/macrophages at the lesion site [44], where these cells phagocytose myelin debris (Figure 6a,b) that would otherwise inhibit axon regeneration [36,76,77]. Interfering with the classical complement pathway by deleting or neutralizing C1q, C3, or CR3 reduces axon regeneration and decreases MBP clearance in the lesion site [44].

However, in other contexts, ablating microglia can promote optic nerve regeneration (Figure 6c,d) [63]. The CX3CR1 antagonist PLX5622 eliminates ~95% of microglia in the retina, resulting in the loss of retinal Aif1 and C1qa expression within 14 days after ONI [44]. However, we observed only a partial reduction in Aif1 and C1qa and an increase in CR3+ monocytes at the injury site, suggesting a compensatory mechanism by which monocytes may infiltrate into a newly vacant microglial niche [44]. This enhanced CR3+ monocyte infiltration at the injury site is slow to resolve, thereby increasing myelin debris clearance and enhancing axon regeneration in several pro-regenerative treatment paradigms. Together, these results suggest that microglia and immune cells serve multiple and often opposing roles within complex and highly dynamic disease pathologies, highlighting the need for a more thorough understanding of how neuroimmune interactions shape disease pathology and for the development of new tools and therapies to differentially modulate pro-degenerative vs. pro-regenerative subpopulations or signals.

## 10. Neuroinflammation and RGC-Intrinsic Regulators of Survival and Axon Regeneration: Synergistic Effects

**Figure 7:**
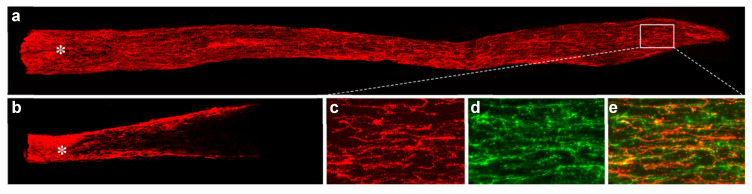
Full-length optic nerve regeneration. (**a**) Intraocular inflammation combined with CPT-cAMP and virally mediated PTEN deletion enables RGCs to regenerate axons the full length of the optic nerve in 8–10 weeks. Axons are labeled by intraocular injection of cholera toxin B fragment (CTB, red). (**b**) Control optic nerve in case treated with intraocular Zymosan and a control virus. (**c**–**e**) Enlargements of area within rectangle show overlap in immunostaining fibers for CTB (**c**, red) and the growth-associated protein GAP43 (**d**, green). Asterisks show lesion site [79].

An important advance in the field of CNS repair was the discovery that modulating RGCs’ intrinsic response to injury can induce considerable axon regeneration. As noted earlier, deletion of Pten, a lipid- and protein phosphatase that suppresses signaling through the PI3 kinase-Akt pathway, enables aRGCs and a small number of other RGCs to regenerate axons through the injured optic nerve [17,78]. Combining Pten deletion with Zymosan and a cAMP analog induces nearly 10 times the level of optic nerve regeneration as any of the individual treatments alone, and, with time, enables some RGCs to regenerate axons into the brain and form synapses in appropriate target areas (Figure 7) [15,79]. mTOR (mammalian target of rapamycin), a central regulator of cell growth, is an important downstream target of PI3 kinase, and disinhibiting mTOR or upregulating a proximate upstream regulator (cRheb1) also promotes optic nerve regeneration, albeit to a lesser extent than Pten deletion [78]. Manipulating cRheb is reported to synergize with increased RGC activity to enable some RGCs to regenerate axons into central target areas [80].

SOCS3 (Suppressor of cytokine signaling) represses Jak-STAT signaling, the transduction pathway activated by CNTF and related factors (e.g., LIF, IL6), and its deletion in RGCs, like that of Pten, promotes optic nerve regeneration. Socs3 deletion also enables recombinant CNTF, which otherwise has little effect on its own, to augment regeneration beyond the level achieved with SOCS3 deletion alone [23]. Combining double deletion of Pten and Socs3 with CNTF treatment allows many axons to regenerate the full extent of the optic nerve and into the brain, albeit into largely inappropriate areas [81,82]. With long survival times, however, some of these axons become able to drive postsynaptic responses in the suprachiasmatic nucleus [83].

By virtue of regulating cells’ program of gene expression, transcription factors represent another key determinant of RGC survival and regenerative capacity. Various members of the Krüppel-like family (KLF) of transcription factors regulate axon outgrowth in RGCs and other neurons [20,84,85]. Deletion of Klf4 promotes a modest level of axon regeneration [84], largely by de-repressing signaling through the Jak-STAT pathway [86]; yet, overexpressing Klf4 along with the transcription factors Oct4 and Sox2 restores immature DNA methylation and transcriptome patterns in RGCs and promotes axon regeneration after optic nerve injury [87]. Deletion of another Krüppel-like TF, Klf9, promotes greater levels of axon regeneration after optic nerve injury than Klf4 deletion [88], an effect that is enhanced even further by combining Klf9 deletion with intraocular Zn^2+^ chelation [89].

One unbiased approach to identifying “master regulators” of the regenerative state is to identify transcription factors that regulate the expression of regeneration-associated genes (RAGs). Using whole-transcriptome RNA sequencing, we identified differentially expressed genes (DEGs) associated with a strong regenerative state in RGCs (Pten deletion combined with the inflammation-associated protein Ocm and a cAMP analog) compared to untreated RGCs. Analysis of cis-regulatory regions of these DEGs predicted the transcriptional and epigenetic repressor REST (NRSF) to be a major repressor of RAGs, a prediction borne out in studies showing that Rest deletion or expression of a dominant-negative Rest mutant promotes considerable axon regeneration and enhances RGC survival after optic nerve injury [90].

Regarding other important cell-intrinsic pathways, Dual leucine zipper kinase (DLK; MAP3K12) and Leucine zipper kinase (LZK; MAP3K13) have been found to be key mediators of RGC cell death after injury [91,92,93]. DLK/LZK activation by axonal injury triggers a retrograde kinase signaling cascade involving MKK4/7 and JNK1-3 that elevates the expression and/or activation of transcription factors that include cJUN, ATF2, MEF2A, and SOX11 [92]. Yet, while repression of DLK and LZK is highly neuroprotective, their deletion suppresses axon regeneration induced by either Pten deletion [91] or Zymosan plus a cAMP analog [94]. Thus, although inhibition of DLK and LZK greatly prolongs RGC survival, this strategy may be insufficient to halt the visual decline in optic neuropathies. Further investigation into the gene regulatory networks downstream of DLK and LZK may enable us to determine if the networks that regulate cell survival vs. axon regeneration diverge such that more focused manipulations might improve both RGC survival and axon regeneration [94]. Newer research from Welsbie and colleagues shows that, unlike DLK and LZK, germinal cell kinase IV (GCK IV) kinases suppress both RGC survival and axon regeneration, and that their deletion in mice is both neuroprotective and amplifies the effects of Pten deletion in promoting optic nerve regeneration [95].

## 11. Prospectives

Transcriptomic comparisons of successfully regenerating RGCs and non-regenerating “bystanders”, single-cell sequencing, large-scale screening of transcription factors and protein kinases, and other modern methods are steadily identifying new therapeutic candidates to enhance RGC survival and axon regeneration after optic nerve injury [92,93,95,96,97,98], lending hope to the possibility that robust RGC neuroprotection and axon regeneration may become a reality in the next 5–10 years. With this goal in sight, the field will also now need to focus on ways to restore at least crude image vision, including the myelination of regenerating axons [99,100] and navigation of regenerating axons to appropriate relay centers in the di- and mesencephalon, where they would need to establish synaptic connections that enable a topographic representation of visual space.

## 12. Conclusions

Although inflammation can have severely negative effects in certain ocular diseases, sterile inflammation in the eye promotes RGC survival and axon regeneration after optic nerve injury through the expression of immune-derived factors that include Ocm, SDF1, the chemokine CCL5, which mediates most of the effects of CNTF gene therapy, and by enhanced phagocytic activity within the optic nerve. As inflammation is an inevitable feature of neural damage, one important goal will be to learn how to modulate the immune response to optimize outcome; a second goal is to identify additional immune-derived factors that can enhance RGC survival and these cells’ ability to form connections with central target areas. Finally, no single approach has yet proven to be a “silver bullet” for restoring visual connections after optic nerve injury, pointing to the likely continuing need for combinatorial treatments to improve functional outcome after optic nerve damage.
